# Interaction Between Lipoprotein(a) and Other Lipid Molecules: A Review of the Current Literature

**DOI:** 10.3390/biom15020162

**Published:** 2025-01-22

**Authors:** Hesham Sheashaa, Hana Mousa, Mohammed Tiseer Abbas, Juan M. Farina, Kamal Awad, Milagros Pereyra, Isabel G. Scalia, Nima Baba Ali, Niloofar Javadi, Nadera N. Bismee, Sogol Attaripour Esfahani, Omar Ibrahim, Fatmaelzahraa Abdelfattah, Ramzi Ibrahim, Mahmoud Abdelnabi, Chadi Ayoub, Reza Arsanjani

**Affiliations:** Department of Cardiovascular Medicine, Mayo Clinic, Phoenix, AZ 85054, USA; sheashaa.hesham@mayo.edu (H.S.); mousa.hana@mayo.edu (H.M.); abbas.mohammedtiseer@mayo.edu (M.T.A.); farina.juanmaria@mayo.edu (J.M.F.); awad.kamal@mayo.edu (K.A.); pereyra.milagros@mayo.edu (M.P.); scalia.isabel@mayo.edu (I.G.S.); babaali.nima@mayo.edu (N.B.A.); javadi.niloofar@mayo.edu (N.J.); bismee.naderanaquib@mayo.edu (N.N.B.); attaripouresfahani.sogol@mayo.edu (S.A.E.); ibrahim.omar3@mayo.edu (O.I.); abdelfattah.fatmaelzahraa@mayo.edu (F.A.); ibrahim.ramzi@mayo.edu (R.I.); abdelnabi.mahmoud@mayo.edu (M.A.); ayoub.chadi@mayo.edu (C.A.)

**Keywords:** lipoprotein(a), interaction, risk factor, mortality, cardiovascular, atherosclerosis

## Abstract

Lipoprotein(a) [Lp(a)] is a well-established causal risk factor for cardiovascular diseases (CVDs), as reported by multiple Mendelian randomization studies and large epidemiological studies. When elevated Lp(a) is combined with other risk factors, most notably elevated low-density lipoprotein cholesterol (LDL-C), a synergistic atherogenic effect has been reported. However, the current literature is conflicting regarding how Lp(a) interacts in the context of controlled LDL-C levels (e.g., <70 mg/dL) and whether reducing LDL-C can modify the atherogenic effect of Lp(a). In some studies, elevated Lp(a) was still significantly associated with a higher risk of cardiovascular events, despite controlled levels of LDL-C. In contrast, multiple studies have reported attenuation of the cardiovascular risk mediated by elevated Lp(a) with lower LDL-C levels. Moreover, the relationship between Lp(a) and triglycerides, high-density lipoprotein, and very low-density lipoprotein remains unclear. In this literature review, we summarize and discuss the current evidence regarding the interactions between Lp(a) and other lipid molecules, how they contribute to the pathogenesis of CVD, and future perspectives, particularly in the current era where promising targeted Lp(a)-lowering therapies are under development.

## 1. Introduction

### 1.1. What Is Lipoprotein(a)?

**Figure 1:**
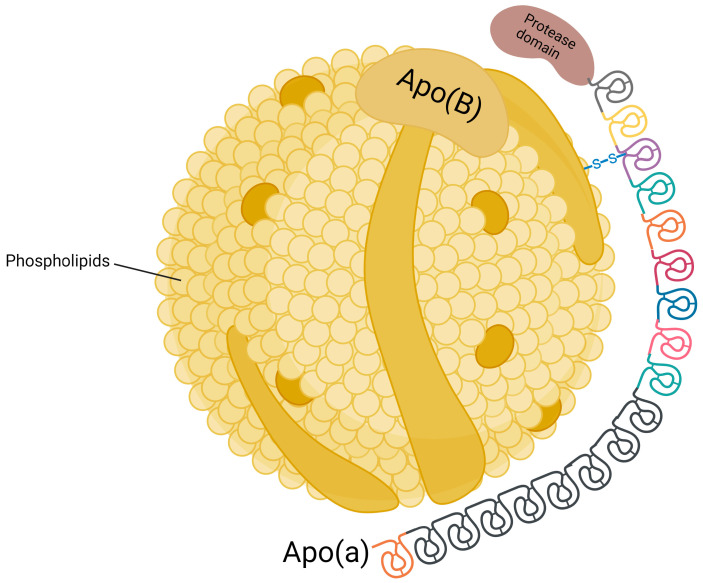
Lipoprotein(a) structure including low-density lipoprotein core with apolipoprotein(B) attached to apolipoprotein(a) by a disulfide bond.

Lipoprotein(a) [Lp(a)] was first described in 1963 by Kåre Berg [1]. It is synthesized in the liver and consists of a low-density lipoprotein (LDL)-like molecule and a unique glycoprotein called apolipoprotein(a) [apo(a)] [2] (Figure 1). Apo(a) has “kringles” which are triple-loop structures that are stabilized by three disulfide bonds [3]. The apoB-100 portion of the LDL molecule is covalently linked to one of these kringles [4].

The serum concentration of Lp(a) is predetermined genetically with minimal environmental influence and is highly variable among individuals [5]. The *LPA* gene on chromosome 6q27 which codes for apo(a) is the major locus controlling Lp(a) concentrations and levels can range from <0.1 mg/dL to >200 mg/dL [3]. The alleles of *LPA* show co-dominant expression [6] and the KIV-2 domain of the *LPA* gene shows significant copy number variation (CNV). Each KIV-2 element can show repetitions of up to 40 times which, in turn, highly influences apo(a) size and Lp(a) concentrations [7]. Apo(a) size is inversely correlated with plasma Lp(a) concentration; this may be due to more difficult protein folding, transport, and secretion of large isoforms [8]. Two possible different apo(a) isoform sizes may be carried by a single individual, one from each allele on chromosome 6, and the allele coding the smaller isoform size will be predominantly expressed [9].

The exact physiological function of Lp(a) is still unknown [3]. A loss of function variant in Austrian and Finnish populations due to a splice site mutation showed no difference in morbidity or mortality [10]. Various functions have been suggested, such as contributing to wound healing and tissue repair, as multiple in vitro studies have shown positive interaction between Lp(a) and components of the extracellular matrix [11]. Additionally, possible involvement in the fibrinolytic and coagulation system has been proposed. There is a high sequence homology between the structure of the *LPA* gene and the plasminogen gene [12]. Apo(a) itself does not have any significant fibrinolytic activity. In fact, it may hinder the activation of plasminogen to plasmin either via competitive inhibition of plasmin or inhibition of plasminogen activators, which, in turn, may aggravate the risk of thrombosis [13,14].

Elevated serum Lp(a) (≥50 mg/dL) is estimated to be present in around 1.4 billion people worldwide [15]. The European Society of Cardiology (ESC) currently recommends Lp(a) plasma measurement once in all adults [16] and both the American College of Cardiology (ACC)/American Heart Association (AHA) and ESC/European Atherosclerosis Society (EAS) recommend its use as a risk enhancer in cardiovascular (CV) risk assessment [17,18]. Lp(a) was shown to be an independent risk factor for numerous CV conditions [19] and may in fact confer an additive effect on CV disease (CVD) risk mediated by low-density lipoprotein—cholesterol (LDL-C) [20]. Although novel therapies under development could reduce Lp(a) levels by up to 80% through alteration of gene expression [21,22,23], most currently approved dyslipidemia drugs have little proven efficacy as regards Lp(a) reduction [24]. Therefore, current guidelines recommend intensive CV risk factor control, including aggressive control of LDL-C, to address the increased risk conferred by high Lp(a) levels [18,25].

Studying lipoprotein interactions is critical as it has been shown that certain interactions can augment the CVD risk while others can mitigate it. For example, the magnitude of CVD risk mediated by elevated Lp(a) when LDL-C is controlled and whether there is a synergistic effect when both are elevated is still a subject of debate. In addition, a proven negative association exists between Lp(a) and triglycerides (TG). However, it is not known to what extent this can influence CV outcomes. Furthermore, there is scarce evidence regarding a potential interaction between Lp(a) and high-density lipoprotein (HDL).

Thus, we aim, in this review, to summarize and discuss the current evidence involving the interaction between Lp(a) and other lipid parameters, mainly LDL-C, and their effect on clinical outcomes.

### 1.2. Measurement of Lp(a)

Accurate Lp(a) measurement is important in clinical practice as it can add to atherosclerotic cardiovascular disease (ASCVD) algorithms, thereby enhancing CVD risk prediction [26]. Lp(a) contains 30–45% cholesterol and this cholesterol content is factored into the reported LDL-C levels [27]. The most common method for measuring Lp(a) is via immunoassays, which use antibodies specific to apo(a) to identify Lp(a) particles [28]. However, Lp(a) has a unique structure that may create certain pitfalls when measuring it. As previously mentioned, the repetitions of the KIV-2 domain in Lp(a) are predominantly responsible for apo(a) size and, in turn, Lp(a) concentration.

Since there is high homology between the KIV-2 repeats, this poses a unique problem when using antibody assays where antibodies may recognize the repetitive motif more than once making molar measurements particularly difficult [29]. Polyclonal antibodies are frequently used which can recognize different epitopes and, as a result, can underestimate or overestimate Lp(a) levels [18]. The use of calibrators has mitigated this issue since each measurement is set in relation to the calibrator and subsequent measurements are adjusted in relation to the calibrator [29]. However, measurement is still challenging due to the multiple genetic variations in apo(a) size, variability in commercial kits, different calibration protocols, and antibody reactivity [28].

Different methods for Lp(a) measurement are used, each with its own advantages and disadvantages. Such methods include radial immunodiffusion (RID), electroimmunoassay, radioimmunoassay (RIA), enzyme-linked immunosorbent assay (ELISA), immunoturbidimetry, nephelometry, dissociation-enhanced lanthanide fluorescent immunoassay (DELFIA), particle concentration fluorescence immunoassay (PCFIA), and electrophoretic and immunofixation electrophoresis [30], with the most commonly used being the nephelometric or immunoturbidimetric assays [29]. Non-antibody-based techniques, such as mass spectrometry, are also being considered to provide more reliable and highly effective measurements compared to the platforms used in clinical laboratories today [22].

Another limitation of traditional methods for measuring Lp(a) concentrations is the storage effect, which results in a decrease in immunoreactivity over time. A significant reduction of up to 75% in Lp(a) levels has been observed after 600 days of storage [31]. In contrast, modern assays have demonstrated substantial improvements, showing only a 4.83% reduction in Lp(a) concentrations after 25 months of storage [29].

Another challenge that may contribute to the inaccuracy occurs when converting Lp(a) values reported in mass units (mg/dL) to its equivalent in nmol/L and vice versa [32]. The National Lipid Association (NLA) supports the use of molar measurements over mass units when reporting Lp(a) because mass units depend on the size of Lp(a) isoforms, although both are valid options [33]. Due to the reasons outlined above, it is not possible to directly convert results based on polyclonal antibodies to molar units. However, in some cases, approximate conversion may be made by comparing results to apo(a) isoform insensitive assays. The most recent EAS consensus statement on Lp(a) specifically advises against such conversions [18].

In the context of these potential inaccuracies, the International Federation of Clinical Chemistry Standardization Group is currently working to standardize Lp(a) assays worldwide and express Lp(a) concentration in SI units [34].

## 2. Lipoprotein(a) and LDL

### 2.1. Correlation Between Serum Lp(a) and LDL-C

Some studies found a statistically significant positive correlation between Lp(a) and LDL-C [19,35,36], while others have failed to observe such correlation [37,38,39,40,41]. Afshar et al. analyzed 939 patients from the GENESIS-PRAXY prospective cohort study of premature atherosclerosis and observed that Lp(a) was significantly associated with serum LDL-C in young acute coronary syndrome patients (*p* < 0.001). This correlation was stronger at higher Lp(a) level, but, at an LDL-C cutoff value of <3.5 mmol/L, this association with Lp(a) became markedly attenuated [36].

### 2.2. Is Lp(a) Associated with Increase Cardiovascular Risk When LDL-C Is Controlled?

#### 2.2.1. Evidence from Observational Studies

Numerous observational studies were conducted to assess the extent of the CVD risk mediated by elevated Lp(a) and whether this risk may be modified by serum LDL-C levels with conflicting results [42,43,44,45,46,47,48,49,50] (Table 1).

An analysis of the Multi-Ethnic Study of Atherosclerosis (MESA) study was conducted, which included 4585 individuals who were free from ASCVD at baseline. It provided evidence that increased levels of Lp(a) carried an increased risk of incident ASCVD regardless of LDL-C levels at baseline. Interestingly, it was also found that there was no increase in ASCVD risk in patients with elevated LDL-C > 100 mg/dL and Lp(a) < 50 mg/dL [51]. In another primary prevention study, The Prospective Cardiovascular Münster (PROCAM) Study, von Eckardstein et al. found that Lp(a) was an independent risk factor for CVD regardless of LDL level [52]. The Precocious Coronary Artery Disease (PROCARDIS) study was a case-control study investigating the association between multiple single nucleotide polymorphisms (SNPs) and the risk of coronary artery disease (CAD). Two independent SNP variants, rs10455872 and rs3798220, were associated with elevated CAD risk mainly through their effects on Lp(a) levels. No differences were observed in the odds of CAD among subgroups with high Lp(a) levels and different LDL-C concentrations [53].

On the other hand, multiple other observational studies have shown discordant findings [36,54,55,56,67,68]. For instance, in an analysis of the PRIME cohort by Luc et al., a significant interaction was observed between Lp(a) and LDL-C when the cohort was stratified by LDL-C levels. The relative risk of CAD events in patients with high Lp(a) (≥33 mg/dL) was increased significantly only in the subjects with elevated levels of LDL-C (≥143 mg/dL) [57]. In an analysis of the EPIC-Norfolk population study and Copenhagen City Heart study on 26,102 individuals in a primary prevention setting, similar findings were observed. Both LDL-C and Lp(a) were risk factors for CAD, except for individuals with low LDL-C levels (<2.5 mmol/L) where Lp(a) associated risk was mitigated [58]. Similar findings were observed in the large prospective Women’s Health Study of 27,791 initially healthy women. The authors found that the risk of CVD was highest among women with both elevated Lp(a) > 90th percentile and LDL-C. However, when stratification was performed based on the LDL-C median, Lp(a) failed to exert a significant atherosclerotic effect when LDL-C levels were below the median, even at the highest Lp(a) quintile [59].

In the secondary prevention setting, several studies showed a dependence of Lp(a) on high LDL-C to exert an atherogenic effect [60,61,62]. A prospective observational study involving 12,604 patients by Yoon et al. studied the association between elevated Lp(a) and composite CV outcomes among patients who had previously undergone percutaneous coronary intervention (PCI). Higher Lp(a) concentration resulted in a higher risk of recurrent CV events in patients with LDL > 70 mg/dL but not LDL ≤ 70 mg/dL [61]. In an analysis based on the Essen Coronary Artery Disease (ECAD) Registry involving 4941 patients who underwent PCI, no association was observed between high Lp(a) levels and adverse outcomes when LDL-C was controlled [63]. In a retrospective cohort study by Mahmoud et al., which included 878 patients who underwent PCI and reached their target LDL-C (<70 mg/dL), the authors observed that there was no significant difference between Lp(a) groups (<50 and ≥50 mg/dL) in terms of survival probabilities of major adverse cardiovascular events (MACE) or all-cause mortality [64].

Furthermore, the interaction between Lp(a) and LDL-C on recurrence of stroke or transient ischemic attack (TIA) has been investigated. Xu et al. conducted a prospective cohort study on 9899 patients with previous stroke/TIA. There was a significant association between elevated Lp(a) and stroke recurrence among patients with high corrected LDL-C. Such association was insignificant at low corrected LDL-C levels (<55 mg/dL) [65].

#### 2.2.2. Evidence from Intervention Studies

The evidence from intervention studies is also conflicting [69,70,71,72,73,74,75,76] (Table 2). A recent meta-analysis by Bhatia et al., including 27,658 patients pooled from six placebo-controlled statin trials, was performed to assess the relationship between elevated LDL-C and Lp(a) levels on the risk of fatal or nonfatal CAD events, stroke, or any coronary or carotid revascularization. It was found that the risk of ASCVD was associated with increased Lp(a) in both statin and placebo-treated patients. The risk of ASCVD in statin-treated patients with high Lp(a) levels remained elevated across all quartiles regardless of achieved LDL-C levels [70]. A previous meta-analysis of seven statin trials also reported similar results [71].

In the FOURIER trial, 25,096 patients with established ASCVD were randomized to evolocumab, a proprotein convertase subtilisin/kexin type 9 (PCSK9) inhibitor, or placebo. The authors found that the benefit of Lp(a) reduction via evolocumab on CAD death, myocardial infarction (MI), or urgent revascularization was independent of baseline or achieved LDL-C levels. Moreover, the greatest benefit was observed in patients who achieved a reduction in both LDL-C and Lp(a) [72]. This finding was similar in the ODYSSEY OUTCOMES trial, which studied alirocumab, and its subsequent posthoc analysis [73,74]; the benefits of reduction of Lp(a) by alirocumab was independent of the achieved reduction in corrected LDL-C, and that alirocumab provided additional mortality reduction in patients with elevated Lp(a) and controlled LDL-C below 70 mg/dL. Moreover, the AIM-HIGH trial, which studied extended-release niacin (ERN), showed congruent results. The elevated levels of Lp(a) contributed to elevated CVD risk despite achieving target LDL-C via LDL-lowering therapy in both the LDL-lowering therapy plus placebo and LDL-lowering therapy plus ERN arms, notwithstanding the side effects associated with niacin [75].

On the other hand, other trials have shown different results. A meta-analysis of multiple trials (including PEACE, CARE, and PROVE IT–TIMI 22) involving 18,978 patients on the role of Lp(a) in secondary prevention of CAD was performed. The patients who were in the highest quintile of Lp(a) concentration had 40% higher odds of MACE. However, when the study results were stratified on the basis of LDL-C, no association was observed between Lp(a) and MACE in patients with lower levels of LDL-C (<130 mg/dL) [19]. Of note, there was significant in-study heterogeneity. Similarly, in a post-hoc analysis of the Familial Atherosclerosis Treatment Study, Maher et al. studied the outcomes following the reduction of LDL-C on the CVD risk mediated by Lp(a). It was found that a high Lp(a) concentration was associated with more disease severity, faster progression, and a higher event rate when high LDL-C was present. In contrast, high Lp(a) was not atherogenic when the LDL-C level was controlled [77].

### 2.3. Do LDL and Lp(a) Have a Synergistic Effect on CVD Risk?

Multiple studies revealed a synergistic interaction between Lp(a) and LDL-C [66,78,79,80]. Hu et al. conducted a case-control study (1522 cases and 1691 controls) aiming to assess the interaction between Lp(a) and LDL-C on the first acute MI. Using the reference group of LDL-C < 2.6 mmol/L and the first quintile of Lp(a) (≤34 mg/L), it was found that the OR (95% CI) of incident MI for only elevated LDL-C (≥2.6 mmol/L) was 2.66 (1.78–3.98) and the ORs (95% CI) for only elevated Lp(a) at the highest quintile were 2.66 (1.88–3.76). A synergistic effect was observed where the odds of incident MI associated with the elevation of both LDL-C and Lp(a) were higher than the sum of the risks of both alone [79].

Shen et al. observed a synergistic effect between Lp(a) and LDL-C and poor coronary collateral formation among diabetes mellitus (DM) patients with stable CAD and coronary chronic total occlusion. A fourfold higher odds of poor coronary collateral formation (OR: 3.970; 95% CI: 1.918–8.216; *p* < 0.001) was found in patients with high LDL-C and high Lp(a) compared to patients with high LDL-C and low Lp(a) [78].

Kronenberg et al. found a synergistic interaction between Lp(a) and LDL-C on the development of early carotid atherosclerosis among 500 individuals who were free from atherosclerosis at baseline [66]. Armstrong et al. showed that the combination of elevated Lp(a) and LDL-C may be associated with a 6-fold higher odds of angiographically classified CAD (*p* < 0.001) among a cohort of 570 males aged 40–60 years [80].

## 3. Lipoprotein(a) and HDL

Studies examining the interaction between Lp(a) and HDL are scarce. The interaction between Lp(a) and HDL might be clinically relevant as it has been proposed that these two molecules may share a common metabolic pathway. Lp(a) may induce the transformation of HDL to a malfunctioning variant, which may affect its beneficial properties [81]. In addition, it has been shown in murine model studies that both Lp(a) and HDL bind to scavenger receptor class B type I (SR-BI) for metabolism [82]. Some studies have found a positive association between serum Lp(a) and serum HDL-C [83,84,85], while others failed to do so [86].

Al Hageh et al. conducted a cross-sectional study on 268 patients who had undergone coronary angiography. They found that patients with Lp(a) ≥ 30 mg/dL have statistically significant higher mean HDL levels in comparison to those with Lp(a) < 30 mg/dL (45.928 ± 13.507 vs. 41.862 ± 9.607, *p* = 0.006) [84]. In addition, the association between high Lp(a) and CAD was paradoxically stronger in patients with high HDL levels. Patients with elevated Lp(a) were at a significantly higher risk of developing CAD, even in the presence of elevated HDL serum levels, suggesting that elevated Lp(a) may mitigate the protective features of HDL [84]. This could be explained by high systemic and vascular inflammation mediated by elevated Lp(a), which leads to the transformation of HDL to a malfunctioning form, eliminating its protective effects against atherosclerosis [81,84].

Additionally, Konerman et al. conducted a cross-sectional study on 148 individuals to evaluate the relationship between Lp(a), TG, and HDL. They reported that the relationship between Lp(a) and HDL varies with TG levels. At high TG levels, Lp(a) showed a strong correlation with HDL2-C/HDL3-C (beta-coefficient = 1.63; *p* = 0.0004), very low-density lipoprotein (VLDL) (beta-coefficient = −1.33; *p* = 0.009) and TG-related variables (beta-coefficient = 0.603; *p* = 0.02) which suggests that Lp(a), VLDL, TG, and HDL may share common metabolic pathways at high TG levels [87]. No such relationship was observed at low TG levels. Consistent with previous studies, Sharma et al. conducted a cross-sectional study on 121 obese (BMI > 85th percentile) African American children aged 9–11 years. A positive correlation was observed between Lp(a) and HDL-C (r = 0.462; *p* < 0.001), including after the adjustment for other plasma lipoproteins [83].

These various interactions between Lp(a) and HDL are important as they may help to better control CV risk. However, further research is necessary to have a full understanding of this relationship.

## 4. Lipoprotein(a) and VLDL/Triglycerides

The relationship between Lp(a) and TGs/VLDL is clinically relevant as a synthesis of both TGs/VLDL and Lp(a) seems to be interrelated [88]. In addition, both molecules may share common metabolic pathways, and it has been demonstrated that Lp(a) degradation may involve VLDL receptors [89].

The inverse correlation between Lp(a) and TG among individuals with hypertriglyceridemia was first described by Bartens et al. [90]. Multiple subsequent studies have confirmed this relationship [83,90,91,92,93,94,95,96]. Ramos-Cáceres et al. conducted a cross-sectional study on 11,406 individuals to analyze the relationship between TG and Lp(a). In addition to confirming the presence of this inverse relationship, they found that larger VLDL particles and apo E-rich VLDL particles were associated with lower serum Lp(a) [94]. Another study including 1,350,908 individuals from the US evaluated the effects of elevated TG/HDL-C ratio, which is associated with obesity, metabolic syndrome, and insulin resistance, on the remaining lipid profile. A statistically significant inverse relationship between TG/HDL-C and Lp(a) was shown (r = −0.32) [97]. Interestingly, a high TG/HDL-C ratio was associated with a more atherogenic lipid profile, including more non-HDL-C, higher LDL density, and more remnant lipoprotein cholesterol (RLP-C), despite being associated with lower Lp(a) levels. Marco-Benedí et al. showed that the relationship between Lp(a) and TG is not linear, and the negative correlation exists between TG and Lp(a) only when TG levels surpass 300 mg/dL [98]. Furthermore, McConathy et al. showed that this inverse relationship does not seem to be affected by the presence of CAD [92].

To explore possible explanations for this relationship, it was shown that a positive correlation exists between TG and the formation of lipoprotein(a)–triglyceride-rich lipoprotein (Lp(a)–TRL) complexes and that the quantity of Lp(a) in the triglyceride-rich lipoprotein (TRL) varied with TG levels [95]. The Lp(a)–TRL complex may be more readily metabolized than Lp(a) alone, explaining the inverse relationship [93]. Another possible explanation for this association was put forward by Ramos-Cáceres et al., who suggested that larger VLDL particles, the presence of apoE in VLDL, and enrichment of VLDL with TG were associated with a drop in Lp(a) synthesis [94].

Marco-Benedí et al. showed similar findings to support this hypothesis. They stratified the study population into four pathologies that are associated with hypertriglyceridemia via different mechanisms: DM (via peripheral insulin resistance, which affects lipoprotein lipase activity), obesity (via decreased peripheral lipolysis), familial hypercholesterolemia (via decreased hepatic lipoprotein catabolism), and multifactorial combined hyperlipemia (via increased synthesis of VLDL and other TG-rich lipoproteins). The first three conditions showed no inverse correlation with Lp(a) [94]. On the other hand, individuals with multifactorial combined hyperlipemia, a condition associated with increased production and enrichment of VLDL, still showed a strong and significant inverse relationship. Thus, the correlation of Lp(a) and TG seems to depend on the etiology of hypertriglyceridemia. This finding is consistent with the effects seen with cholesteryl ester transfer protein (CETP) inhibitor therapy. The drop in Lp(a) observed with CETP inhibitors may be related to the enrichment of VLDL with TG [94,99]. Finally, future research is required to clarify common mechanisms, enzymes, and receptors involved in the metabolism of Lp(a) and TG.

## 5. Discussion and Future Perspectives

After its discovery in 1963 by Kare Berg, Lp(a) has gained remarkable scientific interest after its role in the development and progression of ASCVD was highlighted. This was confirmed after multiple Mendelian randomization studies, and meta-analyses proved its role as a causal risk factor for ASCVD and aortic stenosis [68,100,101,102].

The atherogenic effects of non-HDL lipoproteins seem to be largely dependent on their oxidized phospholipids (OxPL) content. Lp(a) appears to be the preferred carrier lipoprotein for serum OxPL, even more so compared to LDL [103], and Lp(a) was shown to have a longer half-life in the blood [104]. OxPL interacts with endothelial cells, smooth muscle cells, macrophages, and valve interstitial cells in the vascular milieu to induce the development of atherosclerotic plaques and aortic valve dysfunction and degeneration. Lp(a) shows more avid binding to the extracellular matrix compared to LDL, leading to more cholesterol deposition in the atherosclerotic plaque [68]. Finally, Lp(a) may also pose a prothrombotic risk owing to the structural homology with plasminogen, which may inhibit fibrinolysis, especially in patients with smaller apo (a) isoforms [68].

Sex-specific differences in Lp(a) and CV outcomes have been reported. While it was found that Lp(a) levels showed an increase in both genders with age, women experienced a modest increase around menopause [105]. It was suggested that women may benefit from a repeat Lp(a) measurement around that age [105,106]. In a large general population study, the authors found that, in men and women above or below the age of 50, there was a similar morbidity and mortality risk with elevated Lp(a) and a non-significant interaction effect [105].

**Figure 2:**
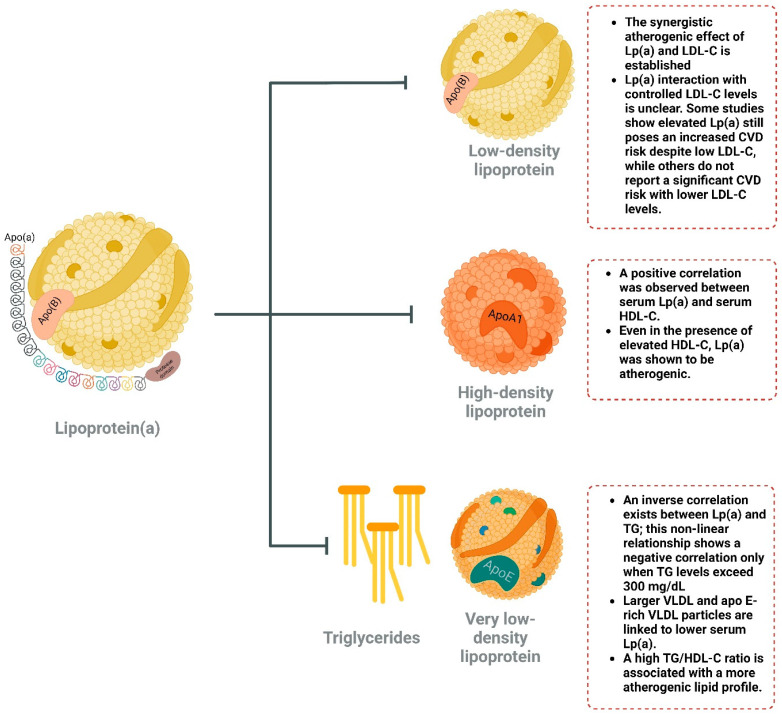
Summary of Lp(a) interactions with different lipid molecules.

It is important to study the interaction between Lp(a) and other lipid molecules, as it was consistently shown that the ASCVD risk mediated by Lp(a) can be profoundly affected by other lipid parameters (Figure 2). There is also a lack of consistent data on the direction of interaction between Lp(a) and other lipids. Multiple large observational and interventional studies have shown that Lp(a) can be an independent risk factor for ASCVD, regardless of the associated serum levels of other lipoproteins or it may depend on the presence of elevated LDL-C to exert its hazardous effects [42,43,44,45,46,47,48,49,50]. These conflicting findings suggest that the impact of Lp(a) may depend on the specific intervention, patient population, and baseline LDL-C levels.

Current phase 2 and 3 trials of specific Lp(a) lowering drugs are underway that will provide more data regarding the true extent of the role of Lp(a) in ASCVD [21,22,23].

It is not clear why several studies consistently concluded that Lp(a) has a negligible effect on CV outcomes when LDL-C is controlled. One possible explanation for this is that Lp(a) catabolism has been shown to be partially dependent on LDL receptors (LDL-R) [107]. In high LDL-C settings, Lp(a) may show enhanced deleterious effects since LDL-C competitively inhibits the LDL-R, amplifying the hazardous effect of Lp(a). Conversely, in the low LDL-C settings, upregulation of the LDL-R enhances the metabolism of Lp(a) and mitigates its deleterious effects [60,64].

Similarly, multiple mechanisms have been suggested that may explain the synergistic atherogenic effects of both Lp(a) and LDL-C. LDL particles may become trapped by the presence of Lp(a) inside the arterial wall via their apoB component. Interaction of oxidized LDL with macrophages may also augment its uptake of Lp(a) particles [108]. Finally, apo(a) may directly interact with other apoB-100-containing lipoproteins resulting in the production of lipoprotein aggregates [109]. Overall, elevation of both Lp(a) and LDL-C was consistently shown to be associated with worse outcomes than when either are elevated on their own. Considering this, current guidelines recommend a once-per-life measurement of serum Lp(a) as well as the use of Lp(a) as a CV risk modifier for more aggressive risk factor control since no current effective therapeutic intervention exists to effectively reduce Lp(a) level [16,17,18]. Future studies are crucial to better understand the relationship between Lp(a) and LDL-C. This is relevant in clinical practice as it will help guide whether it is necessary to reduce Lp(a) in patients with controlled LDL-C or not and to what extent we need to be aggressive with lipid-lowering therapy when elevated Lp(a) is present.

Lp(a) was shown to not only be positively correlated with serum HDL, but it may also have a detrimental effect on its protective features. This correlation suggests that malfunctioning HDL may play a role in the pathophysiology of Lp(a) atherogenesis. Therefore, future therapies that significantly reduce Lp(a) levels might enhance the protective features of HDL. In addition, Lp(a) was consistently shown to be negatively correlated with serum TG and VLDL. It is unclear how this might be clinically relevant. However, it is possible that modification of the composition of VLDL can be used to influence serum Lp(a) in the future.

Finally, although some of the currently available lipid-lowering therapies have not shown sufficient Lp(a) lowering effects, PCSK9 inhibitors have shown reductions in Lp(a) levels, as well as promising results regarding further reduction of CV risk among those with elevated Lp(a) [72,73].

Novel therapies are currently under phase 2 and 3 trials that target the *LPA* gene transcription through the use of small interfering RNAs, or gene translation through the use of antisense oligonucleotides [110], with some capable of achieving up to 80% reduction [21,22,23]. These ongoing and also future trials could help understand the relationship between Lp(a) and LDL-C by including patients with different LDL-C cutoff values or targets prior to enrolment. By doing so, clinical trials of Lp(a) lowering medications could demonstrate that CV risk reduction among patients with elevated Lp(a) may be independent of the baseline or achieved levels of LDL-C and will provide strong evidence for the causal relationship between Lp(a) and ASCVD. These promising treatments may help better understand the relationship between Lp(a) and other lipoproteins and lipid parameters leading to improved CVD risk control and prevention and providing clinically needed intervention to directly affect Lp(a) levels and attenuate its associated CVD risks.

## Figures and Tables

**Table 1:** Observational studies involving Lp(a) and LDL interactions on CVD.

Name of Study	Year	Sample Size	Type of Study	Outcomes
Studies demonstrating that the risk mediated by Lp(a) is independent of the presence of elevated LDL-C				
Ren et al. [47]	2021	988	Retrospective cohort	In post-PCI patients who had well controlled LDL-C (≤1.8 mmol/L) at 1-month follow-up, the presence of elevated Lp(a) was associated with higher risk of MACE (HR: 1.64; 95% CI: 1.04–2.59)
Madsenet al. [48]	2019	3642	Retrospective cohort	Even in cases of low LDL-C (<70 mg/dL), incident MACE among patients with established CVD was higher in the high Lp(a) (≥50 mg/dL) group compared to the low Lp(a) group (<10 mg/dL) (HR: 1.69; 95% CI: 1.16–2.46)
Konishi et al. [49]	2015	411	Prospective cohort	In patients who achieved target LDL-C level (<100 mg/dL) at time of PCI, an increased risk of all-cause mortality and ACS was observed with elevated Lp(a) (HR: 1.68; 95% CI: 1.03–2.70; *p* = 0.04)
Jaeger et al. [50]	2009	120	Retrospective cohort	CAD patients with Lp(a) ≥ 2.14 μmol/l (95th percentile) received lipid lowering therapy followed by apheresis. Apheresis was successful in lowering serum Lp(a) by 73% which was associated with the reduction of MACE irrespective of serum LDL-C before or during apheresis (*p* < 0.001).
Rikhi et al. [51]	2022	4585	Prospective cohort	Elevated Lp(a) was associated with elevated risk of CAD events among individuals free of ASCVD irrespective of LDL-C at baseline (HR for LDL-C ≤ 100 mg/dL and Lp(a) ≥ 50 mg/dL: 1.83; 95% CI: 1.02, 3.27)
PROCAM [52]	2001	788	Prospective cohort	Incident CAD was higher in patients with elevated Lp(a) (≥20 mg/dL) independent of serum LDL-C
PROCARDIS [53]	2009	3145 cases and 3352 controls	Case-control	SNP variants, rs10455872 and rs3798220, were associated with higher CAD odds due to their link with high Lp(a) levels (OR: 1.70; 95% CI: 1.49–1.95) and (OR:1.92; 95% CI: 1.48–2.49) respectively. These findings were consistent across different LDL-C levels.
Studies which failed to demonstrate that the risk mediated by Lp(a) is independent of the presence of elevated LDL-C				
Baldassarre et al. [54]	1996	100 cases and 25 controls	Case-control	Among type II familial hypercholesterolemia patients, elevated Lp(a) (>30 mg/dL) was a risk factor of carotid atherosclerosis only among patients with markedly increased LDL-C (>5.2 mmol/L) (*p* < 0.002)
The Physicians’ Health Study [55]	2004	195 cases and 195 controls (all men)	Case-control	Lp(a) was significantly associated with incident angina when LDL-C was elevated (≥160 mg/dL), but not when LDL-C levels were low.
Cantin et al. [56]	1998	2156	Prospective cohort	No significant increase in the risk of incident IHD was observed when serum Lp(a) was high (≥30 mg/dL) and serum LDL-C was within the first tertile (<3.47 mmol/L) (RR:1.35; 95% CI: 0.57–3.17) compared to the third tertile (≥4.28 mmol/L) (RR: 2.45; 95%CI: 1.26–4.76)
PRIME study [57]	2002	9133	Prospective cohort	The risk of CAD at serum Lp(a) ≥ 33 mg/dL was not statistically significant at the first quartile of LDL-C (<121 mg/dL) (RR: 0.82; 95% CI: 0.28–2.44). The risk becomes statistically significant with higher LDL-C quartiles (RR: 1.58; 95% CI: 1.06–2.40) at the fourth quartile (>163 mg/dL)
Verbeek et al. [58]	2018	26,102	Prospective cohort	Lp(a) was a risk factor for composite cardiovascular outcomes except at low LDL-C levels (<2.5 mmol/L) where the risk mediated by elevated Lp(a) (≥80th percentile) was attenuated. (HR: 1.11; 95% CI: 0.77–1.59)
Women’s Health Study [59]	2006	27,791	Prospective cohort	CVD risk was highest when both LDL-C was ≥the median (121.4 mg/dL) and Lp(a) was ≥the 90th percentile (65.5 mg/dL) among initially healthy women. No significant risk increase was observed when Lp(a) was high and LDL-C was below the median.
Zhu et al. [60]	2022	516	Prospective cohort	In post-PCI patients with a previous ACS, a higher risk of MACE was observed in the high Lp(a) (≥30 mg/dL) group when LDL-C was above a cut-off of 1.4 mmol/L (HR: 1.63; 95% CI: 1.12–2.38, *p* = 0.012). Such association did not exist when LDL-C was <1.4 mmol/L (HR: 0.49; 95% CI: 0.17–1.42, *p* = 0.186)
Yoon et al. [61]	2021	12,064	Prospective cohort	High Lp(a) (>30 mg/dL) was associated with a higher risk of composite CV events post-PCI when LDL-C ≥ 70 mg/dL but not when LDL-C < 70 mg/dL
Cai et al. [62]	2013	832	Prospective cohort	In the high LDL-C group (≥1.8 mmol/L), there was a higher risk of MACE post-PCI in the high Lp(a) subgroup (≥ 30 mg/dL) compared to the low Lp(a) subgroup (6.1% versus 16.6%, *p* = 0.018). In the low LDL-C group (<1.8 mmol/L), no statistically significant difference in the risk of MACE was observed between high and low Lp(a) (16.3% versus 18.5%, *p* = 0.755).
ECAD registry [63]	2023	4941	Prospective cohort	Elevated Lp(a) showed no association with adverse outcomes in post-PCI patients when LDL-C was <100 mg/dL (HR: 1.02; 95% CI: 0.81–1.28); *p* = 0.9]; compared to LDL-C ≥ 100 mg/dL (HR: 1.47; 95% CI: 1.16–1.86); *p* = 0.002)
Mahmoud et al. [64]	2024	878	Retrospective Cohort	In post-PCI patients with well controlled LDL-C (<70 mg/dL), no significant association was observed between high Lp(a) (≥ 50 mg/dL) on the risk of MACE (HR: 1.07; 95% confidence interval: 0.84–1.37) or all-cause mortality (HR: 0.98; 95% CI: 0.74–1.30)
Xu et al. [65]	2022	9899	Prospective cohort	Elevated Lp(a) (≥50 mg/dL) was associated with elevated risk of recurrent stroke (HR: 1.20; 95% CI: 1.02–1.42). This risk was attenuated in patients with concomitant low corrected LDL-C (<55 mg/dL) (HR: 0.92; 95% CI: 0.65–1.30)
Armstrong et al. [66]	1986	428 cases and 142 controls	Case-control	In cases where LDL-C was above the median and Lp(a) > 30 mg/dL, there was 6.0 higher odds of CAD (*p* < 0.001). When the values of LDL-C and total cholesterol were below the median, there was no significant increase in odds of CAD.

Abbreviations: Lp(a): lipoprotein(a); CAD: coronary artery disease; ASCVD: atherosclerotic cardiovascular disease; LDL-C: low-density lipoprotein-cholesterol; HR: hazard ratio; CI: confidence interval; SNP: single nucleotide polymorphism; OR: odds ratio; MACE: major adverse cardiac events; CVD: cardiovascular disease; PCI: percutaneous coronary intervention; ACS: acute coronary syndrome; CV: cardiovascular.

**Table 2:** Intervention studies involving Lp(a) and LDL interactions on CVD.

Name of Study	Year	Number of Individuals	Type	Outcome
Studies demonstrating that the risk mediated by Lp(a) is independent of the presence of elevated LDL-C				
Khera et al. [69]	2014	9612	Post-hoc analysis	Both baseline and on-statin Lp(a) were associated with elevated risk of CAD, independent of serum LDL-C (HR for on statin Lp(a): 1.27; 95% CI: 1.01–1.59; *p* = 0.04)
Bhatia et al. [70]	2024	27,658	Meta-analysis	A significant association was observed between elevated Lp(a) (>50 mg/dL) and CVD risk in statin-treated patients. This finding was consistent across all LDL-C quartiles. In patients with high Lp(a) and in the lowest quartile of achieved LDL-C level, the CVD risk remained elevated (HR: 1.38; 95% CI: 1.06–1.79)
Willeit et al. [71]	2018	29,069	Meta-analysis	Elevated Lp(a) (≥50 mg/dl) remained a residual risk predictor of CVD, even when LDL-C is adequately controlled with statin treatment.
FOURIER trial [72]	2019	25,096	RCT	The benefit of Lp(a) reduction via evolocumab in decreasing CVD risk was independent of baseline or achieved LDL-C levels
ODYSSEY OUTCOMES trial [73]	2020	18,924	RCT	Benefits of reduction of Lp(a) on composite CV outcomes by alirocumab was independent of the achieved reduction in corrected LDL-C
Schwartz et al. [74]	2021	4351	Post-hoc analysis	Patients with recent ACS and controlled LDL-C on statins had a significant reduction in MACE with alirocumab when Lp(a) was ≥13.7 mg/dL (adjusted HR:0.68; 95% CI: 0.52–0.90)
AIM-HIGH trial [75]	2013	3414	RCT	Elevated levels of Lp(a) contributed to elevated CVD risk despite achieving target LDL-C via LDL-lowering therapy in both study arms (LDL-lowering therapy plus placebo vs. LDL-lowering therapy plus ERN)
Studies which failed to demonstrate that the risk mediated by Lp(a) is independent of the presence of elevated LDL-C				
O’Donoghue [19]	2014	18,978	Meta-analysis	There was a significant increase in risk of MACE among patients with baseline CAD and the highest quintile of Lp(a). This became statistically insignificant in a subgroup analysis where only studies with LDL-C < 130 mg/dL were included (OR: 1.20; 95% CI: 0.90–1.60; *p* = 0.21)
Familial atherosclerosis treatment study [77]	1995	146	Post-hoc analysis	In men with CAD and high LDL-C, a strong association existed in the placebo group between Lp(a) and CAD severity at baseline and follow-up. In the statin group patients who achieved target LDL reduction, Lp(a) was no longer associated with CV risk
Familial Hypercholesterolemia Regression Study (FHRS) [76]	1997	39	RCT	There was no improvement in outcomes from the reduction of Lp(a) when LDL-C is controlled among patients with heterozygous familial hypercholesterolemia, either through apheresis or pharmacologically

Abbreviations: Lp(a): lipoprotein(a); CVD: cardiovascular disease; LDL-C: low-density lipoprotein-cholesterol; HR: hazard ratio; CI: confidence interval; ERN: extended-release niacin; ACS: acute coronary syndrome; MACE: major adverse cardiac events; CAD: coronary artery disease; CV: cardiovascular.

## Data Availability

No new data were created or analyzed in this study. Data sharing is not applicable to this article.

## Abbreviations

The following abbreviations are used in this manuscript:

ACC	American College of Cardiology
ACS	Acute coronary syndrome
AHA	American heart association
Apo(a)	Apolipoprotein(a)
ASCVD	Atherosclerotic cardiovascular disease
BMI	Body mass index
CAD	Coronary artery disease
CETP	Cholesteryl ester transfer protein
CI	Confidence interval
CNV	Copy number variation
CV	Cardiovascular
CVD	Cardiovascular disease
DELFIA	Dissociation-enhanced lanthanide fluorescent immunoassay
DM	Diabetes mellitus
EAS	European atherosclerosis society
ELISA	Enzyme-linked immunosorbent assay
ERN	Extended-release niacin
ESC	European society of cardiology
HDL	High-density lipoprotein
HR	Hazard ratio
LDL-C	Low-density lipoprotein cholesterol (LDL-C)
Lp(a)	Lipoprotein(a)
LP(a)–TRL	Lipoprotein(a)–triglyceride-rich lipoprotein
MACE	Major adverse cardiovascular events
MI	Myocardial infarction
NLA	National lipid association
OR	Odds ratio
OxPL	Oxidized phospholipids
PCFIA	Particle concentration fluorescence immunoassay
PCI	Percutaneous coronary intervention
PCSK9	Proprotein convertase subtilisin/kexin type 9
RID	Radial immunodiffusion
RID	Radioimmunoassay
RLP-C	Remnant lipoprotein cholesterol
SNP	Single nucleotide polymorphisms
SR-BI	Scavenger receptor class B type I
TG	Triglycerides
TIA	Transient ischemic attack
TRL	Triglyceride-rich lipoprotein
VLDL	Very low-density lipoprotein

## References

[1] Berg K. (1963). A New Serum Type System in Man—The Lp System. Acta Pathol. Microbiol. Scand..

[2] Utermann G., Weber W. (1983). Protein composition of Lp(a) lipoprotein from human plasma. FEBS Lett..

[3] Schmidt K., Noureen A., Kronenberg F., Utermann G. (2016). Structure, function, and genetics of lipoprotein (a). J. Lipid Res..

[4] Guevara J., Spurlino J., Jan A.Y., Yang C.Y., Tulinsky A., Prasad B.V., Gaubatz J.W., Morrisett J.D. (1993). Proposed mechanisms for binding of apo[a] kringle type 9 to apo B-100 in human lipoprotein[a]. Biophys. J..

[5] Boerwinkle E., Leffert C.C., Lin J., Lackner C., Chiesa G., Hobbs H.H. (1992). Apolipoprotein(a) gene accounts for greater than 90% of the variation in plasma lipoprotein(a) concentrations. J. Clin. Investig..

[6] Ober C., Nord A.S., Thompson E.E., Pan L., Tan Z., Cusanovich D., Sun Y., Nicolae R., Edelstein C., Schneider D.H. (2009). Genome-wide association study of plasma lipoprotein(a) levels identifies multiple genes on chromosome 6q. J. Lipid Res..

[7] Haibach C., Kraft H.G., Kochl S., Abe A., Utermann G. (1998). The number of kringle IV repeats 3–10 is invariable in the human apo(a) gene. Gene.

[8] Lanktree M.B., Anand S.S., Yusuf S., Hegele R.A., Investigators S. (2010). Comprehensive analysis of genomic variation in the LPA locus and its relationship to plasma lipoprotein(a) in South Asians, Chinese, and European Caucasians. Circ. Cardiovasc. Genet..

[9] Parish S., Hopewell J.C., Hill M.R., Marcovina S., Valdes-Marquez E., Haynes R., Offer A., Pedersen T.R., Baigent C., Collins R. (2018). Impact of Apolipoprotein(a) Isoform Size on Lipoprotein(a) Lowering in the HPS2-THRIVE Study. Circ. Genom. Precis. Med..

[10] Lim E.T., Wurtz P., Havulinna A.S., Palta P., Tukiainen T., Rehnstrom K., Esko T., Magi R., Inouye M., Lappalainen T. (2014). Distribution and medical impact of loss-of-function variants in the Finnish founder population. PLoS Genet..

[11] van der Hoek Y.Y., Sangrar W., Cote G.P., Kastelein J.J., Koschinsky M.L. (1994). Binding of recombinant apolipoprotein(a) to extracellular matrix proteins. Arterioscler. Thromb..

[12] McLean J.W., Tomlinson J.E., Kuang W.J., Eaton D.L., Chen E.Y., Fless G.M., Scanu A.M., Lawn R.M. (1987). cDNA sequence of human apolipoprotein(a) is homologous to plasminogen. Nature.

[13] Spence J.D., Koschinsky M. (2012). Mechanisms of lipoprotein(a) pathogenicity: Prothrombotic, proatherosclerotic, or both?. Arterioscler. Thromb. Vasc. Biol..

[14] Koschinsky M.L., Marcovina S.M. (2004). Structure-function relationships in apolipoprotein(a): Insights into lipoprotein(a) assembly and pathogenicity. Curr. Opin. Lipidol..

[15] Enas E.A., Varkey B., Dharmarajan T.S., Pare G., Bahl V.K. (2019). Lipoprotein(a): An independent, genetic, and causal factor for cardiovascular disease and acute myocardial infarction. Indian Heart J..

[16] Mach F., Baigent C., Catapano A.L., Koskinas K.C., Casula M., Badimon L., Chapman M.J., De Backer G.G., Delgado V., Ference B.A. (2020). 2019 ESC/EAS Guidelines for the management of dyslipidaemias: Lipid modification to reduce cardiovascular risk. Eur. Heart J..

[17] Grundy S.M., Stone N.J., Bailey A.L., Beam C., Birtcher K.K., Blumenthal R.S., Braun L.T., de Ferranti S., Faiella-Tommasino J., Forman D.E. (2019). 2018 AHA/ACC/AACVPR/AAPA/ABC/ACPM/ADA/AGS/APhA/ASPC/NLA/PCNA Guideline on the Management of Blood Cholesterol: A Report of the American College of Cardiology/American Heart Association Task Force on Clinical Practice Guidelines. J. Am. Coll. Cardiol..

[18] Kronenberg F., Mora S., Stroes E.S.G., Ference B.A., Arsenault B.J., Berglund L., Dweck M.R., Koschinsky M., Lambert G., Mach F. (2022). Lipoprotein(a) in atherosclerotic cardiovascular disease and aortic stenosis: A European Atherosclerosis Society consensus statement. Eur. Heart J..

[19] O’Donoghue M.L., Morrow D.A., Tsimikas S., Sloan S., Ren A.F., Hoffman E.B., Desai N.R., Solomon S.D., Domanski M., Arai K. (2014). Lipoprotein(a) for risk assessment in patients with established coronary artery disease. J. Am. Coll. Cardiol..

[20] Vuorio A., Watts G.F., Schneider W.J., Tsimikas S., Kovanen P.T. (2020). Familial hypercholesterolemia and elevated lipoprotein(a): Double heritable risk and new therapeutic opportunities. J. Intern. Med..

[21] Tsimikas S., Viney N.J., Hughes S.G., Singleton W., Graham M.J., Baker B.F., Burkey J.L., Yang Q., Marcovina S.M., Geary R.S. (2015). Antisense therapy targeting apolipoprotein(a): A randomised, double-blind, placebo-controlled phase 1 study. Lancet.

[22] Viney N.J., van Capelleveen J.C., Geary R.S., Xia S., Tami J.A., Yu R.Z., Marcovina S.M., Hughes S.G., Graham M.J., Crooke R.M. (2016). Antisense oligonucleotides targeting apolipoprotein(a) in people with raised lipoprotein(a): Two randomised, double-blind, placebo-controlled, dose-ranging trials. Lancet.

[23] Tsimikas S., Karwatowska-Prokopczuk E., Gouni-Berthold I., Tardif J.C., Baum S.J., Steinhagen-Thiessen E., Shapiro M.D., Stroes E.S., Moriarty P.M., Nordestgaard B.G. (2020). Lipoprotein(a) Reduction in Persons with Cardiovascular Disease. N. Engl. J. Med..

[24] Awad K., Mikhailidis D.P., Katsiki N., Muntner P., Banach M., Lipid and Blood Pressure Meta-Analysis Collaboration (LBPMC) Group (2018). Effect of Ezetimibe Monotherapy on Plasma Lipoprotein(a) Concentrations in Patients with Primary Hypercholesterolemia: A Systematic Review and Meta-Analysis of Randomized Controlled Trials. Drugs.

[25] Reyes-Soffer G., Ginsberg H.N., Berglund L., Duell P.B., Heffron S.P., Kamstrup P.R., Lloyd-Jones D.M., Marcovina S.M., Yeang C., Koschinsky M.L. (2022). Lipoprotein(a): A Genetically Determined, Causal, and Prevalent Risk Factor for Atherosclerotic Cardiovascular Disease: A Scientific Statement From the American Heart Association. Arterioscler. Thromb. Vasc. Biol..

[26] Nurmohamed N.S., Kaiser Y., Schuitema P.C.E., Ibrahim S., Nierman M., Fischer J.C., Chamuleau S.A.J., Knaapen P., Stroes E.S.G. (2022). Finding very high lipoprotein(a): The need for routine assessment. Eur. J. Prev. Cardiol..

[27] Yeang C., Clopton P.C., Tsimikas S. (2016). Lipoprotein(a)-cholesterol levels estimated by vertical auto profile correlate poorly with Lp(a) mass in hyperlipidemic subjects: Implications for clinical practice interpretation of Lp(a)-mediated risk. J. Clin. Lipidol..

[28] Wieringa G. (2000). Lipoprotein(a): What’s in a measure?. Ann. Clin. Biochem..

[29] Kronenberg F. (2022). Lipoprotein(a) measurement issues: Are we making a mountain out of a molehill?. Atherosclerosis.

[30] Heydari M., Rezayi M., Ruscica M., Jpamialahamdi T., Johnston T.P., Sahebkar A. (2023). The ins and outs of lipoprotein(a) assay methods. Arch. Med. Sci. Atheroscler. Dis..

[31] Craig W.Y., Poulin S.E., Forster N.R., Neveux L.M., Wald N.J., Ledue T.B. (1992). Effect of sample storage on the assay of lipoprotein(a) by commercially available radial immunodiffusion and enzyme-linked immunosorbent assay kits. Clin. Chem..

[32] Cegla J., France M., Marcovina S.M., Neely R.D.G. (2021). Lp(a): When and how to measure it. Ann. Clin. Biochem..

[33] Koschinsky M.L., Bajaj A., Boffa M.B., Dixon D.L., Ferdinand K.C., Gidding S.S., Gill E.A., Jacobson T.A., Michos E.D., Safarova M.S. (2024). A focused update to the 2019 NLA scientific statement on use of lipoprotein(a) in clinical practice. J. Clin. Lipidol..

[34] Cobbaert C.M., Althaus H., Begcevic Brkovic I., Ceglarek U., Coassin S., Delatour V., Deprez L., Dikaios I., Dittrich J., Hoofnagle A.N. (2021). Towards an SI-Traceable Reference Measurement System for Seven Serum Apolipoproteins Using Bottom-Up Quantitative Proteomics: Conceptual Approach Enabled by Cross-Disciplinary/Cross-Sector Collaboration. Clin. Chem..

[35] Palmeira A.C., Leal A.A., de Medeiros N Ramos N., de Alencar F Neto J., Simoes M.O., Medeiros C.C. (2013). Lipoprotein (a) and cardiovascular risk factors in children and adolescents. Rev. Paul. Pediatr..

[36] Afshar M., Pilote L., Dufresne L., Engert J.C., Thanassoulis G. (2016). Lipoprotein(a) Interactions with Low-Density Lipoprotein Cholesterol and Other Cardiovascular Risk Factors in Premature Acute Coronary Syndrome (ACS). J. Am. Heart Assoc..

[37] Saeedi R., Li M., Allard M., Frohlich J. (2014). Marked effects of extreme levels of lipoprotein(a) on estimation of low-density lipoprotein cholesterol. Clin. Biochem..

[38] Li K.M., Wilcken D.E., Dudman N.P. (1994). Effect of serum lipoprotein(a) on estimation of low-density lipoprotein cholesterol by the Friedewald formula. Clin. Chem..

[39] Bustanji Y., Barham N., Abu-Rish E.Y., Alhyari A., Albustanji B., Alnajjar M., Abu-Irmaileh B., El-Huneidi W., Abu-Gharbieh E., Mohammad M. (2022). Clinical investigation of lipoprotein (a) levels in type 2 diabetics for cardiovascular diseases prediction and prognosis. Horm. Mol. Biol. Clin. Investig..

[40] Mayyas F., Bani Omar E. (2022). Plasma lipoprotein (a) and tissue plasminogen activator are associated with increased risk of atherosclerotic cardiovascular disease. Heliyon.

[41] Jacob E.O., McIntyre A.D., Wang J., Hegele R.A. (2024). Lipoprotein(a) in Familial Hypercholesterolemia. CJC Open.

[42] Emerging Risk Factors C., Erqou S., Kaptoge S., Perry P.L., Di Angelantonio E., Thompson A., White I.R., Marcovina S.M., Collins R., Thompson S.G. (2009). Lipoprotein(a) concentration and the risk of coronary heart disease, stroke, and nonvascular mortality. JAMA.

[43] Ariyo A.A., Thach C., Tracy R., Cardiovascular Health Study I. (2003). Lp(a) lipoprotein, vascular disease, and mortality in the elderly. N. Engl. J. Med..

[44] Jauhiainen M., Koskinen P., Ehnholm C., Frick M.H., Manttari M., Manninen V., Huttunen J.K. (1991). Lipoprotein (a) and coronary heart disease risk: A nested case-control study of the Helsinki Heart Study participants. Atherosclerosis.

[45] Cantin B., Despres J.P., Lamarche B., Moorjani S., Lupien P.J., Bogaty P., Bergeron J., Dagenais G.R. (2002). Association of fibrinogen and lipoprotein(a) as a coronary heart disease risk factor in men (The Quebec Cardiovascular Study). Am. J. Cardiol..

[46] Schaefer E.J., Lamon-Fava S., Jenner J.L., McNamara J.R., Ordovas J.M., Davis C.E., Abolafia J.M., Lippel K., Levy R.I. (1994). Lipoprotein(a) levels and risk of coronary heart disease in men. The lipid Research Clinics Coronary Primary Prevention Trial. JAMA.

[47] Ren Y., Pan W., Li X., Wang S., Lv H., Yu Y., Wang M., Xia Y., Yin D. (2022). The Predictive Value of Lp(a) for Adverse Cardiovascular Event in ACS Patients with an Achieved LDL-C Target at Follow Up After PCI. J. Cardiovasc. Transl. Res..

[48] Madsen C.M., Kamstrup P.R., Langsted A., Varbo A., Nordestgaard B.G. (2020). Lipoprotein(a)-Lowering by 50 mg/dL (105 nmol/L) May Be Needed to Reduce Cardiovascular Disease 20% in Secondary Prevention: A Population-Based Study. Arterioscler. Thromb. Vasc. Biol..

[49] Konishi H., Miyauchi K., Kasai T., Tsuboi S., Ogita M., Naito R., Sai E., Fukushima Y., Katoh Y., Okai I. (2015). Impact of lipoprotein(a) as residual risk on long-term outcomes in patients after percutaneous coronary intervention. Am. J. Cardiol..

[50] Jaeger B.R., Richter Y., Nagel D., Heigl F., Vogt A., Roeseler E., Parhofer K., Ramlow W., Koch M., Utermann G. (2009). Longitudinal cohort study on the effectiveness of lipid apheresis treatment to reduce high lipoprotein(a) levels and prevent major adverse coronary events. Nat. Clin. Pract. Cardiovasc. Med..

[51] Rikhi R., Hammoud A., Ashburn N., Snavely A.C., Michos E.D., Chevli P., Tsai M.Y., Herrington D., Shapiro M.D. (2022). Relationship of low-density lipoprotein-cholesterol and lipoprotein(a) to cardiovascular risk: The Multi-Ethnic Study of Atherosclerosis (MESA). Atherosclerosis.

[52] von Eckardstein A., Schulte H., Cullen P., Assmann G. (2001). Lipoprotein(a) further increases the risk of coronary events in men with high global cardiovascular risk. J. Am. Coll. Cardiol..

[53] Clarke R., Peden J.F., Hopewell J.C., Kyriakou T., Goel A., Heath S.C., Parish S., Barlera S., Franzosi M.G., Rust S. (2009). Genetic variants associated with Lp(a) lipoprotein level and coronary disease. N. Engl. J. Med..

[54] Baldassarre D., Tremoli E., Franceschini G., Michelagnoli S., Sirtori C.R. (1996). Plasma lipoprotein(a) is an independent factor associated with carotid wall thickening in severely but not moderately hypercholesterolemic patients. Stroke.

[55] Rifai N., Ma J., Sacks F.M., Ridker P.M., Hernandez W.J., Stampfer M.J., Marcovina S.M. (2004). Apolipoprotein(a) size and lipoprotein(a) concentration and future risk of angina pectoris with evidence of severe coronary atherosclerosis in men: The Physicians’ Health Study. Clin. Chem..

[56] Cantin B., Gagnon F., Moorjani S., Despres J.P., Lamarche B., Lupien P.J., Dagenais G.R. (1998). Is lipoprotein(a) an independent risk factor for ischemic heart disease in men? The Quebec Cardiovascular Study. J. Am. Coll. Cardiol..

[57] Luc G., Bard J.M., Arveiler D., Ferrieres J., Evans A., Amouyel P., Fruchart J.C., Ducimetiere P., Group P.S. (2002). Lipoprotein (a) as a predictor of coronary heart disease: The PRIME Study. Atherosclerosis.

[58] Verbeek R., Hoogeveen R.M., Langsted A., Stiekema L.C.A., Verweij S.L., Hovingh G.K., Wareham N.J., Khaw K.T., Boekholdt S.M., Nordestgaard B.G. (2018). Cardiovascular disease risk associated with elevated lipoprotein(a) attenuates at low low-density lipoprotein cholesterol levels in a primary prevention setting. Eur. Heart J..

[59] Suk Danik J., Rifai N., Buring J.E., Ridker P.M. (2006). Lipoprotein(a), measured with an assay independent of apolipoprotein(a) isoform size, and risk of future cardiovascular events among initially healthy women. JAMA.

[60] Zhu L., Zheng J., Gao B., Jin X., He Y., Zhou L., Huang J. (2022). The correlation between lipoprotein(a) elevations and the risk of recurrent cardiovascular events in CAD patients with different LDL-C levels. BMC Cardiovasc. Disord..

[61] Yoon Y.H., Ahn J.M., Kang D.Y., Lee P.H., Kang S.J., Park D.W., Lee S.W., Kim Y.H., Han K.H., Lee C.W. (2021). Association of Lipoprotein(a) With Recurrent Ischemic Events Following Percutaneous Coronary Intervention. JACC Cardiovasc. Interv..

[62] Cai A., Li L., Zhang Y., Mo Y., Li Z., Mai W., Zhou Y. (2013). Baseline LDL-C and Lp(a) elevations portend a high risk of coronary revascularization in patients after stent placement. Dis. Markers.

[63] Dykun I., Kampf J., Rassaf T., Mahabadi A.A. (2023). Interaction between elevated lipoprotein(a) and LDL cholesterol on mortality risk in patients with coronary artery disease. Eur. J. Prev. Cardiol..

[64] Mahmoud A.K., Awad K., Farina J.M., Abbas M.T., Ali N.B., Abdalla H.M., Badr A., Elahi M.A., Pereyra M., Scalia I.G. (2024). Controlled low-density lipoprotein cholesterol attenuates cardiovascular risk mediated by elevated lipoprotein(a) after percutaneous coronary intervention. Coron. Artery Dis..

[65] Xu J., Hao X., Zhan R., Jiang X., Jin A., Xue J., Cheng A., Liu J., Lin J., Meng X. (2022). Effect of Lipoprotein(a) on Stroke Recurrence Attenuates at Low LDL-C (Low-Density Lipoprotein) and Inflammation Levels. Stroke.

[66] Kronenberg F., Kronenberg M.F., Kiechl S., Trenkwalder E., Santer P., Oberhollenzer F., Egger G., Utermann G., Willeit J. (1999). Role of lipoprotein(a) and apolipoprotein(a) phenotype in atherogenesis: Prospective results from the Bruneck study. Circulation.

[67] Hopkins P.N., Wu L.L., Hunt S.C., James B.C., Vincent G.M., Williams R.R. (1997). Lipoprotein(a) interactions with lipid and nonlipid risk factors in early familial coronary artery disease. Arterioscler. Thromb. Vasc. Biol..

[68] Nordestgaard B.G., Chapman M.J., Ray K., Boren J., Andreotti F., Watts G.F., Ginsberg H., Amarenco P., Catapano A., Descamps O.S. (2010). Lipoprotein(a) as a cardiovascular risk factor: Current status. Eur. Heart J..

[69] Khera A.V., Everett B.M., Caulfield M.P., Hantash F.M., Wohlgemuth J., Ridker P.M., Mora S. (2014). Lipoprotein(a) concentrations, rosuvastatin therapy, and residual vascular risk: An analysis from the JUPITER Trial (Justification for the Use of Statins in Prevention: An Intervention Trial Evaluating Rosuvastatin). Circulation.

[70] Bhatia H.S., Wandel S., Willeit P., Lesogor A., Bailey K., Ridker P.M., Nestel P., Simes J., Tonkin A., Schwartz G.G. (2024). Independence of Lipoprotein(a) and Low-Density Lipoprotein Cholesterol-Mediated Cardiovascular Risk: A Participant-Level Meta-Analysis. Circulation.

[71] Willeit P., Ridker P.M., Nestel P.J., Simes J., Tonkin A.M., Pedersen T.R., Schwartz G.G., Olsson A.G., Colhoun H.M., Kronenberg F. (2018). Baseline and on-statin treatment lipoprotein(a) levels for prediction of cardiovascular events: Individual patient-data meta-analysis of statin outcome trials. Lancet.

[72] O’Donoghue M.L., Fazio S., Giugliano R.P., Stroes E.S.G., Kanevsky E., Gouni-Berthold I., Im K., Lira Pineda A., Wasserman S.M., Ceska R. (2019). Lipoprotein(a), PCSK9 Inhibition, and Cardiovascular Risk. Circulation.

[73] Szarek M., Bittner V.A., Aylward P., Baccara-Dinet M., Bhatt D.L., Diaz R., Fras Z., Goodman S.G., Halvorsen S., Harrington R.A. (2020). Lipoprotein(a) lowering by alirocumab reduces the total burden of cardiovascular events independent of low-density lipoprotein cholesterol lowering: ODYSSEY OUTCOMES trial. Eur. Heart J..

[74] Schwartz G.G., Szarek M., Bittner V.A., Diaz R., Goodman S.G., Jukema J.W., Landmesser U., Lopez-Jaramillo P., Manvelian G., Pordy R. (2021). Lipoprotein(a) and Benefit of PCSK9 Inhibition in Patients with Nominally Controlled LDL Cholesterol. J. Am. Coll. Cardiol..

[75] Albers J.J., Slee A., O’Brien K.D., Robinson J.G., Kashyap M.L., Kwiterovich P.O., Xu P., Marcovina S.M. (2013). Relationship of apolipoproteins A-1 and B, and lipoprotein(a) to cardiovascular outcomes: The AIM-HIGH trial (Atherothrombosis Intervention in Metabolic Syndrome with Low HDL/High Triglyceride and Impact on Global Health Outcomes). J. Am. Coll. Cardiol..

[76] Kitano Y., Thompson G.R. (1997). The familial hypercholesterolemia regression study: A randomized comparison of therapeutic reduction of both low-density lipoprotein and lipoprotein(a) versus low-density lipoprotein alone. Ther Apher..

[77] Maher V.M., Brown B.G., Marcovina S.M., Hillger L.A., Zhao X.Q., Albers J.J. (1995). Effects of lowering elevated LDL cholesterol on the cardiovascular risk of lipoprotein(a). JAMA.

[78] Shen Y., Chen S., Dai Y., Wang X.Q., Zhang R.Y., Yang Z.K., Hu J., Lu L., Ding F.H., Shen W.F. (2019). Lipoprotein (a) interactions with cholesterol-containing lipids on angiographic coronary collateralization in type 2 diabetic patients with chronic total occlusion. Cardiovasc. Diabetol..

[79] Hu Y., Tao J.Y., Cai D.P., He Y.M. (2020). Interaction of lipoprotein(a) with low-density lipoprotein cholesterol on first incident acute myocardial infarction. Clin. Chim. Acta.

[80] Armstrong V.W., Cremer P., Eberle E., Manke A., Schulze F., Wieland H., Kreuzer H., Seidel D. (1986). The association between serum Lp(a) concentrations and angiographically assessed coronary atherosclerosis. Dependence on serum LDL levels. Atherosclerosis.

[81] Rosenson R.S., Brewer H.B., Ansell B.J., Barter P., Chapman M.J., Heinecke J.W., Kontush A., Tall A.R., Webb N.R. (2016). Dysfunctional HDL and atherosclerotic cardiovascular disease. Nat. Rev. Cardiol..

[82] Yang X.P., Amar M.J., Vaisman B., Bocharov A.V., Vishnyakova T.G., Freeman L.A., Kurlander R.J., Patterson A.P., Becker L.C., Remaley A.T. (2013). Scavenger receptor-BI is a receptor for lipoprotein(a). J. Lipid Res..

[83] Sharma S., Merchant J., Fleming S.E. (2012). Lp(a)-cholesterol is associated with HDL-cholesterol in overweight and obese African American children and is not an independent risk factor for CVD. Cardiovasc. Diabetol..

[84] Al Hageh C., Chacar S., Ghassibe-Sabbagh M., Platt D.E., Henschel A., Hamdan H., Gauguier D., El Murr Y., Alefishat E., Chammas E. (2023). Elevated Lp(a) Levels Correlate with Severe and Multiple Coronary Artery Stenotic Lesions. Vasc. Health Risk Manag..

[85] Giger J.N., Strickland O.L., Weaver M., Taylor H., Acton R.T. (2005). Genetic predictors of coronary heart disease risk factors in premenopausal African-American women. Ethn. Dis..

[86] Kimm S.Y., Pasagian-Macaulay A., Aston C.E., McAllister A.E., Glynn N.W., Kamboh M.I., Ferrell R.E. (1999). Correlates of lipoprotein(a) levels in a biracial cohort of young girls: The NHLBI Growth and Health Study. J. Pediatr..

[87] Konerman M., Kulkarni K., Toth P.P., Jones S.R. (2012). Evidence of dependence of lipoprotein(a) on triglyceride and high-density lipoprotein metabolism. J. Clin. Lipidol..

[88] Nassir F., Bonen D.K., Davidson N.O. (1998). Apolipoprotein(a) synthesis and secretion from hepatoma cells is coupled to triglyceride synthesis and secretion. J. Biol. Chem..

[89] Argraves K.M., Kozarsky K.F., Fallon J.T., Harpel P.C., Strickland D.K. (1997). The atherogenic lipoprotein Lp(a) is internalized and degraded in a process mediated by the VLDL receptor. J. Clin. Investig..

[90] Bartens W., Rader D.J., Talley G., Brewer H.B. (1994). Decreased plasma levels of lipoprotein(a) in patients with hypertriglyceridemia. Atherosclerosis.

[91] Werba J.P., Safa O., Gianfranceschi G., Michelagnoli S., Sirtori C.R., Franceschini G. (1993). Plasma triglycerides and lipoprotein(a): Inverse relationship in a hyperlipidemic Italian population. Atherosclerosis.

[92] McConathy W.J., Trieu V.N., Koren E., Wang C.S., Corder C.C. (1994). Triglyceride-rich lipoprotein interactions with Lp(a). Chem. Phys. Lipids.

[93] Tholstrup T., Samman S. (2004). Postprandial lipoprotein(a) is affected differently by specific individual dietary fatty acids in healthy young men. J. Nutr..

[94] Ramos-Caceres M., Lamiquiz-Moneo I., Cenarro A., Calmarza P., Marco-Benedi V., Bea A.M., Mateo-Gallego R., Puzo J., Ordovas J.M., Civeira F. (2022). Triglyceride Metabolism Modifies Lipoprotein(a) Plasma Concentration. J. Clin. Endocrinol. Metab..

[95] Gaubatz J.W., Hoogeveen R.C., Hoffman A.S., Ghazzaly K.G., Pownall H.J., Guevara J., Koschinsky M.L., Morrisett J.D. (2001). Isolation, quantitation, and characterization of a stable complex formed by Lp[a] binding to triglyceride-rich lipoproteins. J. Lipid Res..

[96] Walek T., von Eckardstein A., Schulte H., Assmann G. (1995). Effect of hypertriglyceridaemia on lipoprotein (a) serum concentrations. Eur. J. Clin. Investig..

[97] Quispe R., Manalac R.J., Faridi K.F., Blaha M.J., Toth P.P., Kulkarni K.R., Nasir K., Virani S.S., Banach M., Blumenthal R.S. (2015). Relationship of the triglyceride to high-density lipoprotein cholesterol (TG/HDL-C) ratio to the remainder of the lipid profile: The Very Large Database of Lipids-4 (VLDL-4) study. Atherosclerosis.

[98] Marco-Benedi V., Cenarro A., Laclaustra M., Calmarza P., Bea A.M., Vila A., Morillas-Arino C., Puzo J., Mediavilla Garcia J.D., Fernandez Alaman A.I. (2024). Influence of triglyceride concentration in lipoprotein (a) as a function of dyslipidemia. Clin. Investig. Arterioscler..

[99] Thomas T., Zhou H., Karmally W., Ramakrishnan R., Holleran S., Liu Y., Jumes P., Wagner J.A., Hubbard B., Previs S.F. (2017). CETP (Cholesteryl Ester Transfer Protein) Inhibition with Anacetrapib Decreases Production of Lipoprotein(a) in Mildly Hypercholesterolemic Subjects. Arterioscler. Thromb. Vasc. Biol..

[100] Sandholzer C., Saha N., Kark J.D., Rees A., Jaross W., Dieplinger H., Hoppichler F., Boerwinkle E., Utermann G. (1992). Apo(a) isoforms predict risk for coronary heart disease. A study in six populations. Arterioscler. Thromb..

[101] Erqou S., Thompson A., Di Angelantonio E., Saleheen D., Kaptoge S., Marcovina S., Danesh J. (2010). Apolipoprotein(a) isoforms and the risk of vascular disease: Systematic review of 40 studies involving 58,000 participants. J. Am. Coll. Cardiol..

[102] Kamstrup P.R., Tybjaerg-Hansen A., Steffensen R., Nordestgaard B.G. (2009). Genetically elevated lipoprotein(a) and increased risk of myocardial infarction. JAMA.

[103] Koschinsky M.L., Boffa M.B. (2022). Oxidized phospholipid modification of lipoprotein(a): Epidemiology, biochemistry and pathophysiology. Atherosclerosis.

[104] Alhomoud I.S., Talasaz A., Mehta A., Kelly M.S., Sisson E.M., Bucheit J.D., Brown R., Dixon D.L. (2023). Role of lipoprotein(a) in atherosclerotic cardiovascular disease: A review of current and emerging therapies. Pharmacotherapy.

[105] Simony S.B., Mortensen M.B., Langsted A., Afzal S., Kamstrup P.R., Nordestgaard B.G. (2022). Sex differences of lipoprotein(a) levels and associated risk of morbidity and mortality by age: The Copenhagen General Population Study. Atherosclerosis.

[106] Awad K., Mahmoud A.K., Abbas M.T., Alsidawi S., Ayoub C., Arsanjani R., Farina J.M. (2024). Intra-individual Variability in Lipoprotein(a) Levels: Findings from a Large Academic Health System Population. Eur. J. Prev. Cardiol..

[107] Nestel P. (2019). Lipoprotein(a) Removal Still a Mystery. J. Am. Heart Assoc..

[108] Alfthan G., Pekkanen J., Jauhiainen M., Pitkaniemi J., Karvonen M., Tuomilehto J., Salonen J.T., Ehnholm C. (1994). Relation of serum homocysteine and lipoprotein(a) concentrations to atherosclerotic disease in a prospective Finnish population based study. Atherosclerosis.

[109] Haberland M.E., Fless G.M., Scanu A.M., Fogelman A.M. (1992). Malondialdehyde modification of lipoprotein(a) produces avid uptake by human monocyte-macrophages. J. Biol. Chem..

[110] Tomic Naglic D., Manojlovic M., Pejakovic S., Stepanovic K., Prodanovic Simeunovic J. (2023). Lipoprotein(a): Role in atherosclerosis and new treatment options. Biomol. Biomed..

